# Repolarization of Ferroelectric Superlattices BaZrO_3_/BaTiO_3_

**DOI:** 10.1038/s41598-019-55475-2

**Published:** 2019-12-12

**Authors:** A. S. Sidorkin, L. P. Nesterenko, Y. Gagou, P. Saint-Gregoire, E. V. Vorotnikov, A. Yu. Pakhomov, N. G. Popravko

**Affiliations:** 1 Physical Department, Voronezh State University, University sq. 1, 394018 Voronezh, Russia; 2 Laboratoire de Physique de la Matière Condensée, Universite de Picardie Jules Verne, 80039 Amiens, CEDEX France; 3 UFR Sciences and Techniques, University of Toulon, 83041 Toulon, CEDEX France; 4 Sciences and Arts, University of Nimes, 30021 Nimes, CEDEX France

**Keywords:** Materials science, Nanoscience and technology

## Abstract

With the use of the modified Sawyer-Tower scheme and Merz technique, studies were conducted on the repolarization characteristics of ferroelectric (BaZrO_3_/BaTiO_3_) superlattices on monocrystalline MgO substrate. Studies of temperature changes in the dielectric hysteresis loops indicated a sufficiently smooth decrease in spontaneous polarization compared with homogeneous barium titanate near the phase transition temperature of the superlattice. Experimental studies of switched currents have shown that the switching processes in the synthesized superlattices are implemented in two stages: activation motion (“creep” mode) and non-activation motion (slip mode). The presence of the activation switching stage and the numerical estimates show that with high probability, the movement of domain boundaries accomplishes the processes of switching in the studied superlattice. The threshold field separating the stated stages decreases with increasing temperature up to the Curie point of the superlattice, similar to the coercive field. Detection of the non-strictly exponential dependence of the switching current on the reverse field strength in the activation stage was modulated by the dependence with the power-law exponent for the applied electric field. Both techniques indicate that the studied superlattices have a small internal displacement field directed from the superlattice to the substrate.

## Introduction

Presently, artificially created layered materials, such as ferroelectric superlattices, are actively studied both in the fundamental physics of solid-state nanostructures and in applied physical materials science. The creation and study of these structures, representing multilayer structures in the form of sequentially deposited epitaxial layers of various ferroelectric or ferroelectric and dielectric materials, allow for the purposeful formation of materials with adjusted properties, which is essential for practical applications.

The most common application of these materials is the compact low-frequency capacitors with a large specific capacity. The use of the large polarization non-linearity of these materials for dielectric amplifiers, modulators and other controlled devices is very popular^1–4^. The possibility of obtaining the required switching characteristics of these materials for their use in ferroelectric memory devices has attracted particular attention to the switching processes of ferroelectric thin-film structures and superlattices^5–7^.

The presence of a domain structure in ferroelectric superlattices and thin films was observed in experiments using X-ray diffraction^8,9^. The polarization vector changes due to its correlation with deformation. This change leads to shifting of atomic planes consisting of the same atoms in domains with opposite sign, and it was registered using Wulff-Bragg’s condition.

The basic theoretical representations of the change in polarization of ferroelectric superlattices are based on the Landau - Khalatnikov model^10–17^, in which the kinetics of repolarization is determined by the degree of deviation from the equilibrium state and the relaxation time of the order parameter or by the viscosity coefficients for changing the polarization in different layers of the superlattice. Wherein the possible presence of lattice potential relief (dependence of the energy of the domain boundary on the position of its centre relative to atoms in the elementary cell) was not taken into account. Without an assessment of its impact on the change in polarization, it is impossible to determine presumablewaysof repolarization: with or without the participation of the domain structure. The purpose of this work is a more detailed study of the polarization switching in ferroelectric superlattices using the Sawyer-Tower and the Merz methods.

## Materials and Methods

When creating ferroelectric superlattices, the most attention is paid to various combinations of materials of the perovskite group as the initial series of compounds. First, this is due to the relative simplicity of their structure and the properties of these compositions being well understood. To study the switching processes in the present work, we consider a two-layer ferroelectric superlattice of BaZrO_3_/BaTiO_3_ (BZ/BT)^18,19^ with comparable sizes of elementary cells, which causes appearance of induced strains between the components, changing properties of the superlattice. These superlattice consists of 32 recurring layers of BZ and BT, with the thickness of individual layers of barium zirconate being 6.65 nm and that of barium titanate being 6.67 nm, and a period Λ = 13.32 nm applied by pulsed laser deposition on a substrate of monocrystalline magnesium oxide (MgO) (*a*_*MgO*_ = 4.213 Å), oriented with respect to the (001) plane with a sublayer of conductive oxide La_1/2_Sr_1/2_CoO_3_ (LSCO) (*a*_*LSCO*_ = 3.805 Å) as the lower electrode and platinum as the top electrode, with a radius of 0.5 mm.

The specified samples were obtained in a deposition chamber equipped with a 15-kV reflection high-energy electron diffraction (RHEED) system. With its help, the surface quality of the layers composing the superlattices was systematically monitoredat various stages of growth. The RHEED bands obtained from the BZ and BT layers and their azimuth positions showed the ideal epitaxial growth of the cube on the cube:parameters a and b of the superlattice (the cell sizes in the plane parallel to the substrate) are equal to each other within the experimental error, and angle between the axes [100] and [010] is 90°^20^.

X-ray diffraction studies were carried out using a cobalt anode as a source of X-ray radiation with wavelengths λ_α1_ = 1.7889 Å, λ_α2_ = 1.7928 Å (not used), and λ_β_ = 1.6208 Å. These diffraction studies of the superlattices have shown that they are single crystal formations. Its interplanar distance was *a* = 4.084 Å, which does not correspond to either the barium titanate or the barium zirconate compound: the bulk BT at room temperature had a tetragonal structure with dimensions *a*_*BT*_ = 3.992 Å and *c*_*BT*_ = 4.036 Å, and the adjacent BZ layer for the bulk material had a cubic structure with a size *a*_*BZ*_ = 4.192 Å.

To identify the switching patterns of synthesized superlattices, a modified Sawyer-Tower scheme of studying dielectric hysteresis loops with conductivity compensation was used, as well as the Merz method, which accounts for the switching currents caused by bipolar rectangular pulses of the electric field.

The switching current was measured by the voltage drop due to the resistance in series with the sample and recorded on the screen of a two-channel digital oscilloscope (TDS 2075). For this purpose, periodic voltage pulses of the same duration with an amplitude from 50 mV to 8 V and a frequency of 10 kHz were applied to a ferroelectric capacitor with a sample connected in series with a reference resistance equal to 50 Ω. The equidistant (polarizing and repolarizing) bipolar rectangular pulses were produced with the use of an Arbitrary Waveform Generator 2571 with a time increase of the switching voltage of no more than 10 ns. The pulse duration was from 5 to 80 μs.

The value of the switching current was determined by subtracting the discharge of the linear component of the capacitance from the total current pulse. The measured values were the integral switching characteristics: the maximum value of the switching current pulse *i*_*max*_ and the total switching time *τ*_*s*_. The switching time *τ*_*s*_ was defined as the interval between the start of the current pulse and the time at which the switching current falls to 10% of the maximum value *i*_*max*_.

As it was established by Fatuzzo and Merz in 1957, dependencies of the maximum switching current *i*_*max*_ *(Е)* and the reverse switching time on the strength of the switching field are similar in the areas of “weak” and “strong” fields. Therefore, to determine the desired parameters and describe the switching process, you can use any of these two dependencies. We have chosen the dependence for the maximum switching current.

## Results

**Figure 1:**
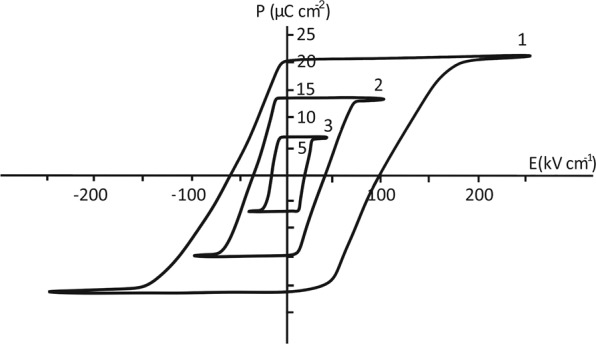
Dielectric hysteresis loop for the BaZrO_3_/BaTiO_3_ superlattice at different temperatures: 1, 2 and 3 correspond to 250 °C, 360 °С and 385 °С, respectively.

The dielectric hysteresis loops of the superlattices, revealed by applying an electric field normal to the substrate at different temperatures, are illustrated in Fig. 1.

According to these studies the synthesized superlattices have a spontaneous polarization in the direction normal to a large surface of superlattices, the value of which is 22.0 μC cm^−2^ at room temperature. The estimation of the coercive field for switching superlattice polarization using the dielectric hysteresis loops showed that, at room temperature, E_c_ = 130 kV cm^−1^.

Note that in contrast to bulk barium titanate, in which the first-order phase transition occurs, in the case of the investigated superlattice, a much smoother decrease in the spontaneous polarization is observed when approaching the temperature at which transition to the nonpolar phase occurs. Indicated changes in the temperature behavior of the polarization can be interpreted as a change in the phase transition from the first type to the second in the ferroelectric superlattices versus bulk barium titanate. For all we know, similar effects have not been observed previously in ferroelectric superlattices. And meanwhile, the same changes in the type of the phase transition were recordered in the calculation work on ferroelectric composites^21^.

**Figure 2:**
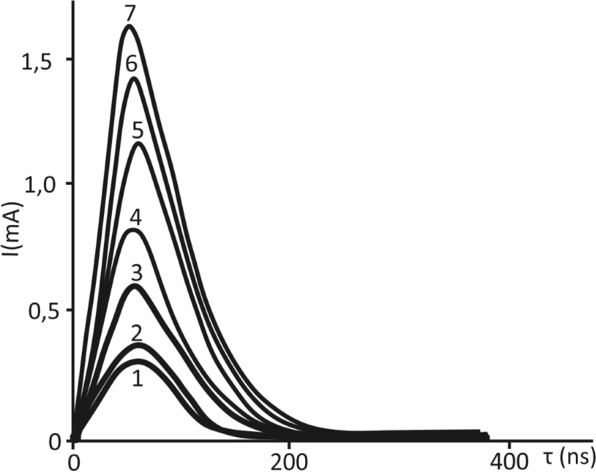
Switching currents of the BaZrO_3_/BaTiO_3_ superlattice for different values of the switching fields: 1, 2, 3, 4, 5, 6 and 7 correspond to 22 kV cm^−1^, 28 kV cm^−1^, 117 kV cm^−1^, 164 kV cm^−1^, 187 kV cm^−1^, 210 kV cm^−1^, and 235 kV cm^−1^, respectively.

**Figure 3:**
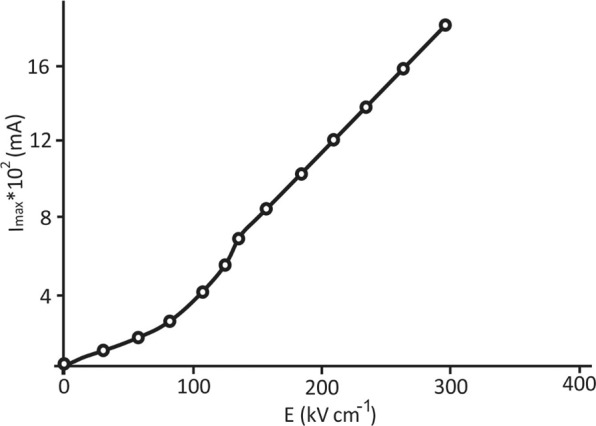
Characteristic dependence of the switching current on the external electric field strength for the BaZrO_3_/BaTiO_3_ superlattice at a temperature of 20 °C.

Figure 2 displays the switching current pulses of the studied superlattice as a function of the total switching time *τ*_*s*_ for different values of the switching fields. Determination on these data the dependencies of the maximum values of the switching currents on the applied field strength shows that these dependencies generally have the form shown in Fig. 3.

According to Fig. 3, the full switching curve, similar to ferroelectric films^22^, has two sections: the initial, so-called “activation” area or the “weak” fields area, where the indicated switching current dependence on the applied field is close to the exponent, and the subsequent linear section, that is, the “strong” fields area or the “slip” area, where the dependence of the maximum switching current is proportional to the field *i*_*max*_ ≈*µPЕs/d* (P – polarization, µ- mobility of domain walls, s - upper electrode area, d – sample thickness).

**Figure 4:**
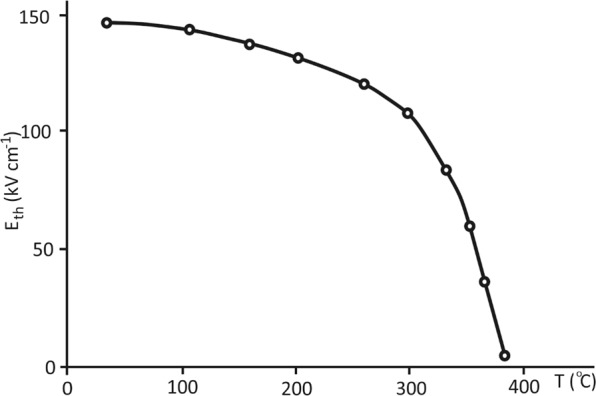
Temperature dependence of the threshold field separating the regions of weak and strong fields using the switching current data for BaZrO_3_/BaTiO_3_ superlattices.

The boundary between the areas of the activation and nonactivation switching regimes, the so-called threshold or critical field E_th_, determined by the switching currents as a field corresponding to the transition of an exponential dependence to a linear one, approximately corresponds to the coercive field determined by the dielectric hysteresis loop. As shown in Fig. 4, this threshold field decreases when approaching the ferroelectric phase transition temperature.

**Figure 5:**
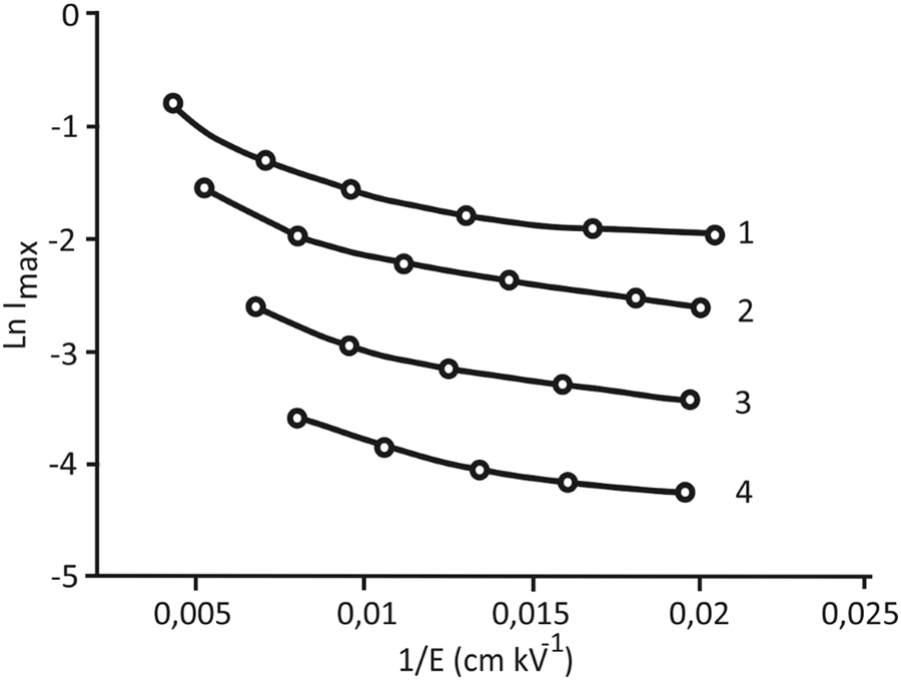
Dependence of the logarithm of the maximum switching current on the inverse field strength for the BaZrO_3_/BaTiO_3_ superlattice in the region of weak fields at different temperatures: 1, 2, 3 and 4 correspond to 65 °C, 120 °C, 230 °C and 350 °C, respectively.

A more detailed examination shows that the behaviour of the switching current in the weak fields areas differs from the usual activation mode. The activation switching regime in the area of “weak” fields is usually described by an exponential law: *i*_*max*_ = *i*_*max∞*_ exp*(−α/E)*, where *α* is an activation field that depends on the temperature. A plot of *ln I*_*max*_ versus the reverse field strength *1/E* (Fig. 5) for the studied structures shows that this dependence of the BaTiO_3_/BaZrO_3_ superlattice is not a straight line.

This means that the simple activation dependence indicated above no longer describes the real dependence of the switching current on the field strength. In this case, it can be described by a more complicated formula in which the power-law exponent is introduced into the above activation dependence: the maximum value of the switching current is proportional to ~ exp*(−α/E*^*μ*^)^23^.

The selection of the power-law exponent μ introduced into the indicated dependence makes it possible to determine its values corresponding to the model function that are most consistent with the experimental dependence. Table 1 shows the values of the matched index μ for different temperatures. In comparison with ferroelectric films^22^, the stated index at room temperature has a much lower value and is practically independent of the temperature.

**Table 1:** Value of the matched index μ for the BaZrO_3_/BaTiO_3_ superlattice for weak fields at different temperatures.

*µ* BaZrO_3_/BaTiO_3_	24 °С	65 °С	120 °С	200 °С	250 °С	300 °С	350 °С
	0,21	0,21	0,20	0,20	0,196	0,19	0,19

**Figure 6:**
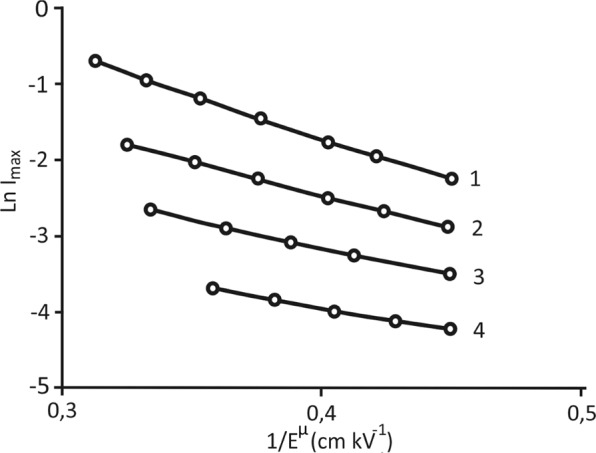
Dependence of the logarithm of the maximum switching current on the inverse field strength, raised to the power of μ, for the BaZrO_3_/BaTiO_3_ superlattice for weak fields at different temperatures: 1, 2, 3 and 4 correspond to 65 °C, 120 °C, 230 °C and 350 °C, respectively.

To determine the “effective activation field” (constant α in the formula *I*_*max*_ ~ exp[−α/*E*^*μ*^], which describes the so-called “creep” processes), the dependence of the logarithm of the maximum value of the switching current ln *I*_*max*_ was constructed based on the inverse field strength exponentiation to amatching degree 1/*E*^*μ*^. As seen from Fig. 6, these dependences are already straight lines, the slopes of which can help one determine the values of the activation fields for different temperatures. The latter are presented in Table 2. As expected, when the superlattice approaches the Curie point, the value of the effective activation field decreases with increasing temperature.

**Table 2:** Value of the activation fields for the BaZrO_3_/BaTiO_3_ superlattice in the weak fields area at different temperatures.

α BaZrO_3_/BaTiO_3_ kV cm^−1^	24 °С	65 °С	120 °С	200 °С	250 °С	300 °С	350 °С
	11,0	10,1	8,8	7,6	6,2	6,1	5,6

**Figure 7:**
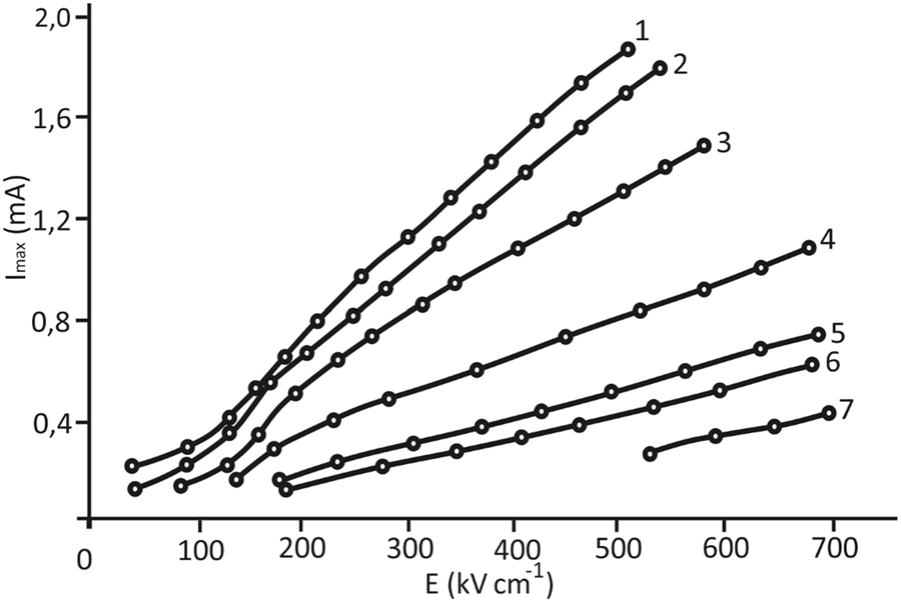
Dependence of the switching current on the external field strength for the BaZrO_3_/BaTiO_3_ superlattice at various temperatures: 1, 2, 3, 4, 5, 6 and 7 correspond to 20 °C, 100 °C, 200 °C, 250 °C, 300 °C, 350 °C and 400 °C, respectively.

After the termination of the “activation” stage in fields greater than the threshold (the area of “strong” fields), the dependence of the maximum switching current on the applied field is a straight line. The performed studies show that in this area (Fig. 7), the incline angle of the field dependence of the switching current decreases with increasing temperature, which indicates a decrease in the mobility of domain walls near thephase transition temperature in non-polar phase due to the growth of the switching time here.

According to the conducted studies, the samples of synthesized superlattices have an internal bias field, which can be determined by the displacement of the dielectric hysteresis loop and by the point of intersection a continuationof the linear section of *i*_*max*_*(E)* dependences with the field axis.

The results of its determination by the two indicated methods approximately coincide and show that at a temperature of 20 °C, the internal bias field is 32 kV cm^−1^ and slightly decreases with increasing temperature. From here, the internal displacement field is directed from the superlattice to the substrate, which can be explained by the flexoelectric deformations of the material deposited on the lower electrode^24^.

### Discussion of the switching nature

The main factors determining the properties of ferroelectric superlattices and the possibility of their change are mechanical stresses caused by the mismatch of sizes of the elementary cells of components of the superlattice which are in contact with each other and with the substrate. The stretching of BaTiO_3_ layers in BaTiO_3_/BaZrO_3_ superlattices due to epitaxial conjugation with BaZrO_3_ layers, having a higher lattice parameter, should stabilize polarization in BaTiO_3_along the substrate.However, as shown in^25^, the presence of structural instability in BaZrO_3_under rotations of octahedra and its competition with ferroelectric instability qualitatively change the situation.

In biaxially compressed BaTiO_3_/BaZrO_3_ ferroelectric superlattices with the strain in the layer plane within 1–3%, the turning of the octahedra leads to an improper ferroelectric phase transition with increasing temperature, and, as a result, to the appearance of a nonzero z-component of polarization. This polarization is summed up with the polarization induced in the barium zirconate layer along the normal to the substrate due to its compression. The indicated total polarization is recordered by observing the dielectric hysteresis loops under the external electric field applied normal to the substrate (Fig. 1). The polarization normal to the substrate with a high probability should lead to the formation of a plane-parallel (Kittel) domain structure, providing repolarization of the superlattices in a weak field.

In principle, repolarization can be carried out by switching the material as a whole or by using the movement of domain boundaries^26–29^.

To answer the question about the possibility of realization in this case the switching of the material as a whole, it is necessary to compare the values of the coercive field actually detected in the experiment with the theoretical estimates of the material switching field as a whole.

The coercive field, corresponding to the switching of the material as a whole, is determined, in thermodynamic theory, from the condition of the extremum of the dependence of the application electric field on polarization:^29^

1 $${E}_{c}=\frac{2}{3\sqrt{3}}\alpha \sqrt{\frac{\alpha }{\beta }}=\frac{4\pi }{3\sqrt{3}}\frac{{P}_{0}}{\varepsilon }$$

where *α* and *β* are the expansion coefficients of the thermodynamic potential in a series in terms of the polarization, *P*_0_ is the spontaneous polarization, and *ε* is the dielectric permittivity of the material. Substituting into (1) the registered value of the spontaneous polarization *P*_0_ ~ 22.0 μC cm^−2^, the value of the dielectric constant at room temperature *ε* ~ 10 ÷ 50^24^, we obtain a coercive field to switch the observed material as a whole that is equal to 2000 kV cm^−1^, which exceeds the experimentally observed coercive field in the material under study.

According to formula (1), the assessment of the coercive field, which determines the possibility of switching the material as a whole, is completely based on the value of the dielectric constant of the material and its polarization obtained on the same sample within the same experiment^24^, which significantly increases the reliability of the conclusion obtained using specified assessment.

In accordance with the evaluation, the presence of the activation area in the regularities of the switching current of the studied ferroelectric superlattices from the field indicates that, with a high probability, the polarization processes are realized by the motion of the domain walls. The activated process in this case is the formation of nuclei on the domain wall stimulated by temperature fluctuations in the applied field with a favourable orientation of the polarization vector. With the value of the external field equal to that of the coercive field, the activation stage ends. In this case, the domain wall no longer senses the restraining switching barrier, and its motion is controlled by the corresponding processes of viscosity, where *i*_*max*_ *≈ const ⋅ E*.

Really, from the point of view of the domain structure, the threshold or critical field for a perfect (defect-free) sample is determined by a field that makes it possible to overcome the Peierls barrier, that is, the difference in the energies of the main and saddle configuration of the domain wall, arising from the coordinate dependence of the energy of the domain wall in the discrete material. The specified field is equal to^29^.

2 $${E}_{c}=\frac{{V}_{0}}{\alpha \,{P}_{0}}$$

where *V*_0_ is the Peierls barrier, *P*_0_ is the spontaneous polarization, and *a* is the size of the unit cell.

Substituting here, found by the experiment, the value of the lattice constant *a* = 4.084 Å, the spontaneous polarization, equal to 22.0 μC cm^−2^, and a value of the lattice barrier on the order of 1 erg cm^−2^, typical for crystals of the barium titanate family^30^, we obtain the theoretical value of the threshold field close to the experimentally observed value of approximately 100 kVcm^−1^. The coincidence of the experimentally observed and estimated values of the threshold field is an additional argument in favour of the domain mechanism of switching of the studied superlattices.

## Conclusions

A study of the switching of ferroelectric barium zirconate/barium zirconate superlattices on a single-crystal magnesium oxide substrate consisting of 32 single-crystal layers with a period of Λ = 13.32 nm has shown that the switching processes of the studied superlattices are carried out in two stages: the “creeping” stage and the sliding stage. The threshold field separating these stages corresponds to the coercive field and decreases with increasing temperature as the temperature approaches the Curie point of the superlattice.

The presence of the activation stage, the theoretical estimates of the coercive field switching of various mechanisms, and their comparison with experiments for the actually observed switching processes indicate that with high probability, the motion of the domain walls causes the switching processes in the studied superlattices. At the pre-threshold stage, the activation process is the nucleation of a new phase on a moving domain wall. Here, the energies of the applied field are still insufficient to overcome the barrier that deters the switching, and an activation fluctuation on the temperature side (lattice vibrations) assists the switching process.

When the applied field reaches a value equal to the coercive field, the lattice barrier no longer affects the domain wall, and its motion is under the control of the corresponding processes of energy dissipation accompanying the movement of the domain boundaries (phonon inhibition, interaction with defects accompanying the motion of the domain wall, etc.).

In the section of the switching current linearly dependent on the field, the inclination angle of the stated current dependence decreases with increasing temperature, which can be explained by a decrease in the mobility of the domain wall due to critical deceleration of the polarization relaxation near the Curie point.

In the studied structures, an internal bias field was estimated from shifting of the dielectric hysteresis loops and from the intersection of the linear segment of the switching current with the field axis,so its value is approximately 32 kV/cm at room temperature and slightly decreases with increasing temperature. The specified internal field for the studied heterostructure substrate, i.e., the multilayer superlattice, is directed from the superlattice to the substrate. The action of the flexoelectric effect explains the emergence of the internal field of the stated direction.

Integral switching characteristics, and hence the speed of the motion of the domain walls in fields less than the threshold value, do not obey a strictly exponential dependence on the field strength. This leads to the appearance of a power-law exponent for the applied electric field μ in the dependence of the switching current (the velocity of the domain walls) on the electric field strength, which is much smaller in comparison with that of thin ferroelectric films^22^ and changes insignificantly when approaching the phase transition point.

In our opinion, the appearance of the power index μ deviated from one, in the exponent of the switching current may be due to not a change in the switching mechanism, but, for example, to a decrease in the average value of the switched polarization with a decrease in polarization near the boundaries of the layers of various materials.
